# Parents are poor at labelling wheeze in children: a cross-sectional study

**DOI:** 10.1186/s12887-016-0616-8

**Published:** 2016-06-23

**Authors:** Shalini Shanmugam, Anna Marie Nathan, Rafdzah Zaki, Kian Eng Tan, Kah Peng Eg, Surendran Thavagnanam, Jessie Anne de Bruyne

**Affiliations:** Department of Paediatrics, University Malaya, 50603 Kuala Lumpur, Malaysia; University Malaya Paediatric and Child Health Research Group, University Malaya, 50603 Kuala Lumpur, Malaysia; Department of Social & Preventive Medicine, Faculty of Medicine, Julius Centre University of Malaya, 50603 Kuala Lumpur, Malaysia

**Keywords:** Asthma, Noisy breathing, Children, Wheeze, Recognise, Stridor

## Abstract

**Background:**

Noisy breathing is a common presenting symptom in children. The purpose of this study is to (a) assess parental ability to label wheeze, (b) compare the ability of parents of children with and without asthma to label wheeze and (c) determine factors affecting parental ability to label wheeze correctly.

**Methods:**

This cross-sectional study in a tertiary hospital in Kuala Lumpur, Malaysia involved parents of children with asthma. Parents of children without asthma were the control group. Eleven validated video clips showing wheeze, stridor, transmitted noises, snoring or normal breathing were shown to the parents. Parents were asked, in English or Malay, “What do you call the sound this child is making?” and “Where do you think the sound is coming from?”

**Results:**

Two hundred parents participated in this study: 100 had children with asthma while 100 did not. Most (71.5 %) answered in Malay. Only 38.5 % of parents correctly labelled wheeze. Parents were significantly better at locating than labelling wheeze (OR 2.4, 95 % CI 1.64–3.73). Parents with asthmatic children were not better at labelling wheeze than those without asthma (OR1.04, 95 % CI 0.59–1.84). Answering in English (OR 3.4, 95 % CI 1.69–7.14) and having older children with asthma (OR 9.09, 95 % CI 3.13–26.32) were associated with correct labelling of wheeze. Other sounds were mislabelled as wheeze by 16.5 % of respondents.

**Conclusion:**

Parental labelling of wheeze was inaccurate especially in the Malay language. Parents were better at identifying the origin of wheeze rather than labelling it. Physicians should be wary about parental reporting of wheeze as it may be inaccurate.

**Electronic supplementary material:**

The online version of this article (doi:10.1186/s12887-016-0616-8) contains supplementary material, which is available to authorized users.

## Background

Noisy breathing is a common presenting symptom, both in acute and chronic respiratory disease. Noises such as wheeze, stridor and transmitted noises are commonly heard, especially in the young [1]. The ability to appreciate and distinguish these different sounds is important as it indicates the level of airway involvement and hence the probable disease pathology.

In multi-ethnic Malaysia, families come from different cultures and socio-demographic backgrounds and speak various languages. Malaysian parents may use different terms to describe various respiratory sounds. For clinicians, an accurate history is essential in making the right diagnosis and providing the best treatment. We are all aware of the shortcomings of medical jargon, when conversing with parents, yet sometimes it is unavoidable. Wheeze is one such commonly term in use.

Sheila McKenzie and colleagues in 2000 and 2001 published 2 studies looking at parental understanding of wheeze and the ability of parents to identify wheeze [2, 3]. They found discordance between parental and physician understanding of wheeze [3]. In their second study, where parents were shown videos of wheeze and other respiratory sounds, one third of them used different words for wheeze and another third mislabelled other respiratory sounds as wheeze [2]. Subsequent studies have made similar findings, illustrating the confusion over the commonly used word, wheeze [4, 5].

We hypothesised that most Malaysian parents would not be able to label wheeze correctly and that parents of children with asthma would be better at labelling wheeze than those of non-asthmatic children.

Hence the aim of this study was to (a) assess the ability of parents at labelling wheeze, (b) compare the ability of parents of children with and without asthma at labelling wheeze and (c) determine factors that affect parental ability at labelling wheeze correctly.

## Methods

### Ethics statement

Ethical approval was obtained from the Research and Ethics Committee, University Malaya Medical Centre (UMMC) (MEC Ref No: 902.16). Written and informed consent in English or Malay was obtained from parents prior to their participation.

### Setting and study design

This was a cross-sectional study carried out in University Malaya Medical Centre (UMMC) a tertiary hospital in Kuala Lumpur, Malaysia. Data was collected prospectively from 1^st^ January 2012 till 30^th^ June 2013. Convenience sampling was used in this study. We included all parents attending the paediatric asthma clinic. Parents of children with noisy breathing other than wheeze, those not fluent in either English or Malay, and those with medical experience were excluded. Parents of children without asthma and other respiratory diseases were recruited from the paediatric wards and served as the control group. Parents were asked to identify type and location of respiratory sounds shown in video clips.

### Video clips and validation

Children with audible respiratory sounds were filmed with a video camera, after consent was obtained. Either a Sony Handycam HDRSR10E or a hand phone with a minimum 8.1mega pixel camera was used to take the video clips. Clips were reviewed by the authors and only those with audible, single respiratory sounds were selected. They were subsequently edited to last a maximum of one minute and eyes were blurred to preserve anonymity of the patients by using Windows Live Movie Maker (Version 1.3.2) and Final Cut Pro software (Version 6.0.6). These clips were then reviewed by the authors and categorised as either normal or showing wheeze, stridor, transmitted noises or snoring.

Qualified paediatricians were selected for the validation process. Video clips were played consecutively and participating paediatricians were required to categorize these noises as normal, wheeze, stridor, snoring or transmitted sounds. Each video clip had only 1 correct response. A correct response of ≥80 % was required to validate each video clip (kappa 0.8). Video clips were categorised into 3 different age groups as shown in Table 1.

**Table 1:** Categorization of the video clips

VIDEO SET 1	VIDEO SET 2	VIDEO SET 3
(Less than 1 year)	(1–5 years)	(6 years and above)
1a. 1 Normal	2a. 1 Normal	3a. 1 Normal
1b. 1 Wheeze	2b. 1 Wheeze	3b. 1 Wheeze
1c. 1 Stridor	2c. 1 Stridor	3c. 1 Snoring
1d. 1 Transmitted sounds	2d. 1 Transmitted sounds	

### Parental viewing of videos

Parents were shown age-appropriate videos in a quiet room using a 14.0 inch computer screen with two external speakers. They could ask to view each clip a maximum of 3 times.

### Questionnaire

Two questions were posed to parents while viewing the video. The first was the “label question” where they were asked “What do you call the sound this child is making?” The answers were open ended to allow parents to use their own words. The second question was the “location question” where they were asked “Where do you think the sound is coming from: the nose, throat or chest?” The responses for the “label” question were collected, checked against reputable dictionaries [6, 7]. Responses were accepted in English or Malay only.

### Definitions

Educational level was sub-grouped into basic if they had primary or secondary education or advanced if they had tertiary education. Asthma was categorised as intermittent if the patient was on ß_2_ agonists alone and persistent if the patient was on any form of preventer medication.

### Statistical analysis

Statistical analysis was done using Statistical Package for the Social Sciences (SPSS) version 16.0. Demographic characteristics of participants were reported in percentage terms (%). Percentages of correct and incorrect answers were compared between groups. Odds Ratio (OR) with 95 % Confidence Interval (CI) was used as the measure of association in this study. The Chi-squared or Fisher’s exact test (where appropriate) was used to test the significance of association between potential risk factors and a correct label for wheeze. The following factors were considered important and were the only factors analysed using binary logistic regression in an attempt to determine independent factors associated with correct labelling of wheeze: respondent (mother or father), age of patient, presence of asthma or not, language used to answer the questionnaire, educational background and severity of asthma. A *p*- value of < 0.05 was considered as significant.

## Results

### Video clips

A total of 38 video clips with various respiratory sounds were obtained. Seventeen were excluded due to poor sound quality or background noise. In the remaining 21, there were 8 videos of wheeze, 3 videos of stridor and 10 other videos (transmitted sounds, normal and snoring).

### Validation

Validation was done in two stages. Video clips were played to an audience of 22 experienced paediatricians from various hospitals in the country. Those with a correct response of ≥80 % (kappa 0.8) were considered valid. Table 1 Out of 21 video clips, 10 were successfully validated but 9 were finally chosen: 2 wheeze, 2 stridor, 2 normal, 1 snoring and 2 transmitted sounds. A second validation was carried out for three new video clips showing normal breathing as we did not have enough video clips showing normal breathing. We were unable to obtain suitable videos of older children with wheeze; hence one video clip (video 3b) from the original validated Asthma Video Questionnaire, International Study of Asthma and Allergies in Childhood (ISAAC), was used with permission from the Wellington Asthma Research Group, University of Otago, New Zealand. This clip had a sensitivity and specificity of 70.0 and 66.2 % respectively [8]. Therefore in total, there were 11 validated videos [9].

All video clips, except for the video from the ISAAC study, are available online as supplementary data (EVideo Additional files 1, 2, 3, 4, 5, 6, 7, 8, 9 and 10).

### Video questionnaire

A total of 200 parents participated in this study: 100 parents had children with asthma while the rest did not. Demographic data of the participants and their children are shown in Table 2. There was no significant difference in baseline characteristics between asthmatics and non-asthmatics. Malays were the majority ethnic group, which is in keeping with our national population demography. Most of the respondents had secondary or tertiary education. Malay was the preferred language of the respondents. As for the asthmatics, most children (*n* = 98, 98 %) were ≥ 1 year old, had persistent asthma (*n* = 76, 76 %) and were living in an urban area (*n* = 82, 82 %).

**Table 2:** Demographic characteristics of children with asthma [*N* = 100] and children without asthma [*N* = 100]

Characteristics	Asthmatics	Controls	*P* value
	*N* (%)	*N* (%)	
Female	35 (35)	45 (45)	0.149
Male	65 (65)	55 (55)	
Race			
Malay	65 (65)	67 (67)	
Chinese	8 (8)	18 (18)	0.057
Indian	24 (24)	14 (14)	
Others	3 (3)	1 (1)	
Age			
Years (median)	6.96	4.24	0.204
(range)	(4.04–11.37)	(1.44–8.75)	
Respondent			
Mother	77 (77)	83 (83)	0.301
Father	20 (20)	16 (16)	
Others	3 (3)	1 (1)	
Respondent’s education level			
Primary	1 (1)	1 (1)	0.56^a^
Secondary	52 (52)	54 (54)	
Tertiary	48 (48)	44 (44)	
Respondent’s role as caregiver			
Main caregiver	95 (95)	96 (96)	1.00^a^
Not main caregiver	5 (5)	4 (4)	
Respondent’s language			
English	33 (33)	24 (24)	0.159
Malay	67 (67)	76 (76)	

^a^Fisher’s exact test

### Primary objective

Only 77 parents (38.5 %) were able to correctly label wheeze. Parents were significantly better at identifying where wheeze originated from than labelling wheeze (OR = 2.44 *p* < 0.001) (Table 3). This was also true for snoring and stridor (OR = 44.68 *p* < 0.001) but not for transmitted noises (Table 3).

**Table 3:** Parental responses to videos showing wheeze, stridor, snoring and transmitted noises

Factors	Correct	Wrong	*P* value	OR	95 % CI
	*N* (%)	*N* (%)			
Wheeze (*n* = 200)					
Label question^a^	77 (38.5)	123 (61.5)			
Location question	121 (60.5)	79 (39.5)	<0.001	2.44	1.64–3.66
Stridor (*n* = 99)					
Label question^a^	1 (1.0)	98 (99.0)			
Location question	31 (31.3)	68 (68.7)	<0.001	44.68	5.96–335.10
Snoring (*n* = 101)					
Label question^a^	45 (44.6)	56 (55.4)			
Location question	8 (7.9)	93 (93.9)	<0.001	0.19	4.11–21.25
Transmitted noises (*n* = 99)					
Label question^a^	55 (55.6)	43 (43.4)			
Location question	41 (41.4)	58 (58.6)	0.04	0.55	0.31–0.97

^a^Reference category

### Secondary objectives

Parents of children with asthma were no better than parents of non-asthmatic children at labelling wheeze (Table 4). English-speaking parents and parents if older children (6 years and above) were better at labelling wheeze. Neither level of parental education nor having a child with asthma improved the ability to correctly label wheeze. Mothers were no better at labelling wheeze than fathers (Table 4).

**Table 4:** Factors affecting correct labelling of wheeze in children by parents

Variables^a^	Correct	Incorrect	Crude	95 % CI	Adjusted OR	Adjusted 95 % CI	*P* Value
	*N* (%)	*N* (%)	OR				
Father^b^	14 (41.2)	20 (58.8)					
Mother	60 (38.5)	96 (61.5)	0.89	0.42–1.94	1.37	0.55–3.37	0.75
Asthma^b^	39 (39.0)	61 (61.0)					
Non-asthma	38 (38.0)	62 (62.0)	0.96	0.54–1.70	1.72	0.85–3.50	0.14
Age of child							
< 6 years^b^	5 (12.5)	35 (87.5)					
≥ 6 years	34 (56.7)	26 (43.3)	5.11	2.73–9.60	7.12	3.63–16.63	<0.001
Language used to answer video questionnaire							
Malay^b^	39 (39.4)	60 (60.6)					
English	38 (69.0)	17 (30.9)	4.76	2.47–9.17	7.12	3.04–16.69	<0.001
Educational Level							
Higher^b^	38 (41.3)	54 (58.7)					
Basic	39 (36.1)	69 (63.9)	0.80	0.45–1.42	1.16	0.65–2.51	0.71
Severity of asthma (*n* = 100)							
Intermittent^b^	9 (37.5)	15 (44.1)					
Persistent	30 (39.5)	46 (60.5)	0.92	0.36–2.37	1.02	0.28–3.65	0.98

^a^Variables included in the model were as follows: respondent (mother or father), age of patient, presence of asthma or not, language used to answer the questionnaire, educational background and severity of asthma

^b^Reference category

Words for wheeze commonly used by parents were “*susah nafas*” (*n* = 31, 21.2 %) and “*tercungap-cungap*” (*n* = 5, 3.4 %) which essentially mean “difficulty in breathing”. Parents also used the word “*kahak*” which means “phlegm” (*n* = 27, 18.8 %). Only one parent described wheeze as whistling in Malay. The proper Malay word to describe wheeze, according to the Kamus Dewan, Iskandar Teuku, Dewan Bahasa dan Pustaka, 1970 dictionary, is “*berdehit*” which was only used by one respondent. Words describing wheeze were used by 33 parents (16.5 %) to describe other respiratory sounds.

**Fig. 1:**
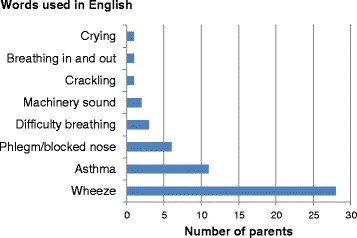
Common English words used by parents to describe wheeze

Of the respondents who answered in English, 49.1 % (*n* = 28) used “wheeze” and 19.3 % (*n* = 11) used “asthma” to describe wheezing. The other terms used to describe wheeze were either incorrect or vague. Nobody used the word “whistling” Fig. 1.

## Discussion

We found that only a third of Malaysian parents correctly labelled wheeze. Parents were significantly better at locating the site where the wheeze came from than labelling it. Parents who had children with asthma were not better than parents of children without asthma, at labelling wheeze. Parents who answered in English and parents of children older than 6 years old were more likely to correctly label wheeze.

This study was inspired by the work of RS Cane and SA McKenzie, who published 2 studies alerting physicians that there is discordance between parental and physician interpretation of wheeze [2, 3]. They found a discrepancy in parental complaints and the clinical findings of doctors, hence resulting in false positive and false negative usage of the word wheeze. In their second study, published in 2001, they found that 59 % of parents correctly labelled wheeze and 30 % of parents called wheeze by another name. Another third called other sounds wheeze [2]. The “overuse” of the word wheeze was also a finding by Lowe et al., who looked at lung function (S_raw_) in children who had never wheezed, in children with parentally reported but unconfirmed wheeze and in those with doctor diagnosed wheeze. He found similar lung function results between the unconfirmed wheezers and those who had never wheezed, indicating that parents tended to overuse the word wheeze [5]. Elphick et al. interviewed parents with children less than 18 months old, both in the community and in hospital, and also found that wheeze was a commonly misused term, especially in parents who have been “institutionalized” [4].

In our study, only 38.5 % of parents correctly labelled wheeze, far worse than in RS Cane’s study. Our findings also differ significantly from others, in that the word wheeze (both in English and Malay respondents), was not overused by parents (16.5 % used wheeze to describe other sounds). Hence, perhaps in populations where English is not the native language, there is underuse of the word wheeze and as such under-recognition of asthma and other wheezing disorders, if doctors diagnose wheezing disorders based on parental reporting of wheeze. This finding also highlights the possible limitation of using written asthma questionnaires in countries where the native language is not English. Culturally-appropriate words to describe wheeze should be used rather than directly translating it. A good example is the ISAAC study, a large international epidemiological study to determine the prevalence of asthma and other related parameters. They too used “whistling sound”, to describe wheeze. This word was used by only one parent in our study [10].

In RS Cane’s first study, the authors developed a video questionnaire and approached parents with wheezy children. Two thirds of parents described wheeze as “difficulty in breathing” and/or “being unwell” and “sound”. It is interesting that in our study, about 30 % of parents described wheeze as difficulty breathing or feeling unwell/tired in Malay, similar to RS Cane’s study. However parents with young children frequently described wheeze as having “phlegm” as most young children who wheeze also have secretions which in turn obstruct the airway and contribute to the wheezing sound. In a small qualitative study that looked at how parents recognize and interpret respiratory symptoms and signs in their child, the author found that parents do not use sound alone but use other cues like how the child looks and behaves when ill. In our study, words like “being unwell” and “difficulty breathing” were also common words used to describe “wheeze” [11].

In Cane and McKenzie’s second study, parents were better at locating than labelling wheeze. Similarly, in our study, parents were significantly better at locating the origin of the wheeze rather than labelling it suggesting that clinicians should use the location of the sound to aid in identifying wheeze.

We then went on to determine factors associated with correct identification of wheeze and intuitively we thought that parents who had wheezing children would do better. Surprisingly, there was no significant difference between parents with and without asthmatic children. Cane et al. also found that parents of children with asthma were no better at labelling wheeze [2]. This could be due to the fact that wheeze is rarely audible without a stethoscope. Severity of asthma and the level of parental education were also not associated with correct labelling of wheeze. Being conversant in English and having older children were significant factors associated with accurate answers. Wheeze in young children is more difficult to recognise, as explained earlier, due to the presence of other extraneous sounds like those related to secretions. That may explain why more parents of older children with asthma labelled wheeze correctly than parents of younger children. Cane et al. also found that translating “wheeze” to other languages was a barrier and hence the difficulty in getting accurate answers is a problem. In the Malay dictionary, “berdehit” is the exact translation of “wheeze”. Only one parent used that word. In the English responses, nobody used the word “whistling” a common word used by doctors to describe wheezing.

Finally, use of video questionnaires may be useful not only in research but also in clinical practice [12]. The next step is to assess the clinical utility and clinical impact of these videos on patient management and outcome.

Limitations of our study are recognized. The quality of the videos may not have been ideal as they were not made in soundproofed rooms. This was unavoidable as these children came in acutely unwell and had to be videoed quickly before the sounds abated. Another major problem which we encountered was trying to get videos of children with pure single respiratory sounds, something that is unusual especially in young children. We also did not enquire about other possible sources of knowledge in parents of children without asthma such as prior experiences that might have lead them to know what wheeze is. Controls i.e. parents of children without asthma, were taken from the same hospital ward and this may not be representative of the community. These parents are more likely to be ‘medicalised’ from exposure in hospital. Finally a larger sample size might have allowed us to investigate other factors that might result in correct identification of wheeze. Our findings may not reflect the knowledge of all Malaysians, as this was done in a tertiary hospital in an urban city in Malaysia.

## Conclusion

Our study re-emphasizes that clinicians should realize the limitation of using the word “wheeze” or “whistling sound” when eliciting a history from parents as many parents, especially if English is not their native language, use other words to describe wheeze. Asking the origin of the sound may be more accurate than using the word wheeze. Malaysian parents do not overuse the word wheeze. Finally, clinicians could use audio segments to help obtain an accurate history of wheezing from parents.

## Additional files

Additional file 1: Video 1a-normal breathing in a child  < 1 year old (MP4 2694 kb) Additional file 2: Video 1b- wheeze in a child  < 1 year old. (MP4 8037 kb) Additional file 3: Video 1c- stridor in a child  < 1 year old. (MP4 5778 kb) Additional file 4: Video 1d- transmitted sounds in a child  < 1 year old. (MP4 5267 kb) Additional file 5: Video 2a- normal breathing in a child 1-5 years old. (MP4 7596 kb) Additional file 6: Video 2b- wheeze in a child 1-5 years old. (MP4 1970 kb) Additional file 7: Video 2c- stridor in a child 1-5 years old. (MP4 3187 kb) Additional file 8: Video 2d- transmitted sounds in a child 1-5 years old. (MP4 2718 kb) Additional file 9: Video 3a-normal breathing in a child >= 6 years old. (MP4 3532 kb) Additional file 10: Video 3c- snoring in a child >= 6 years old. (MP4 3343 kb)
